# Modeling Realistic Geometries in Human Intrathoracic Airways

**DOI:** 10.3390/diagnostics14171979

**Published:** 2024-09-07

**Authors:** Francesca Pennati, Lorenzo Aliboni, Andrea Aliverti

**Affiliations:** Dipartimento di Elettronica, Informazione e Bioingegneria, Politecnico di Milano, 20133 Milan, Italyandrea.aliverti@polimi.it (A.A.)

**Keywords:** airways, geometrical models, imaging, CT, MRI, image processing, segmentation, biomechanical models, pulmonary disease, deep learning

## Abstract

Geometrical models of the airways offer a comprehensive perspective on the complex interplay between lung structure and function. Originating from mathematical frameworks, these models have evolved to include detailed lung imagery, a crucial enhancement that aids in the early detection of morphological changes in the airways, which are often the first indicators of diseases. The accurate representation of airway geometry is crucial in research areas such as biomechanical modeling, acoustics, and particle deposition prediction. This review chronicles the evolution of these models, from their inception in the 1960s based on ideal mathematical constructs, to the introduction of advanced imaging techniques like computerized tomography (CT) and, to a lesser degree, magnetic resonance imaging (MRI). The advent of these techniques, coupled with the surge in data processing capabilities, has revolutionized the anatomical modeling of the bronchial tree. The limitations and challenges in both mathematical and image-based modeling are discussed, along with their applications. The foundation of image-based modeling is discussed, and recent segmentation strategies from CT and MRI scans and their clinical implications are also examined. By providing a chronological review of these models, this work offers insights into the evolution and potential future of airway geometry modeling, setting the stage for advancements in diagnosing and treating lung diseases. This review offers a novel perspective by highlighting how advancements in imaging techniques and data processing capabilities have significantly enhanced the accuracy and applicability of airway geometry models in both clinical and research settings. These advancements provide unique opportunities for developing patient-specific models.

## 1. Introduction

Geometrical models of the airways have revolutionized our understanding of lung diseases, acting as a bridge between theoretical research and clinical application. Initially conceptualized from mathematical frameworks [1,2], these models have evolved to incorporate detailed analyses of lung imagery [3], becoming a potent tool for detecting morphological changes in the airways. These changes often act as early indicators of diseases such as Chronic Obstructive Pulmonary Disease (COPD) [4]. The precise representation of airway geometry has proven indispensable in a variety of research domains, including biomechanical modeling [5], acoustics [6,7], and prediction of particle deposition [8,9,10]. More importantly, these models have directly translated into clinical advancements in the diagnosis and treatment of lung diseases [11,12,13], underscoring their translational value.

The lower respiratory tract, a complex and diverse branching system characterized by irregular dichotomy, exhibits significant inter-variability in higher generations. From the trachea to the alveoli, approximately 23 generations of branches can be identified, each becoming narrower and shorter as the generation level increases. In the 1960s, the first attempts were made to encapsulate the anatomical knowledge of the tracheobronchial tree into models that could consistently represent its complexity [1,2]. These initial models were based on ideal mathematical approaches derived from lung casts or observations of lung sections from cadavers.

However, the advent of new imaging techniques such as computerized tomography (CT) and, to a lesser extent, magnetic resonance imaging (MRI), coupled with increased computing power for data processing, has revolutionized the concept of anatomical modeling of the bronchial tree. These advancements have led to the development of image-based, patient-specific geometries that offer reliable support in diagnosing pathological conditions and monitoring their progression in response to treatment [14,15,16]. This translational impact of geometrical models in clinical practice cannot be overstated. This review aims to provide a comprehensive overview of the methods developed to create an accurate representation of the tracheobronchial tree, ranging from early mathematical models [1,2] to more recent image-based techniques [17,18], with a translational perspective. We will discuss the limitations and challenges encountered in both mathematical and image-based modeling, delve deeper into the applications of these models in various fields, and touch upon future directions. The foundation of image-based modeling, the recent segmentation strategies from CT and MRI scans, and their implications in clinical practice will also be scrutinized.

By providing a chronological review of these models, we aim to offer insights into both the evolution and the potential future of airway geometry modeling with a particular emphasis on their translational impact in the diagnosis and treatment of lung diseases. Our review offers a novel perspective by highlighting how the combination of imaging techniques with mathematical models provides unique opportunities for developing patient-specific models. When integrated with functional imaging, these models can help us comprehend the interplay between structural and functional impairments, making them the most practical means of achieving future clinical applications.

## 2. Mathematical Models

The complexity of the bronchial tree is effectively captured and simplified through mathematical models, which establish a collection of parameters that accurately depict each branch order in isolation as well as in correlation with the hierarchical arrangement of the tree. This section provides a comprehensive overview of the evolution of mathematical models, tracing their development through the groundbreaking geometries presented in the literature (refer to Table 1). For an in-depth exploration of additional mathematical models, please refer to the review provided in [19].

**Table 1:** Seminal geometrical models of the tracheobronchial tree, reported in chronological order.

Mathematical Model	Year	Key Points	Data Source	Number of Generations	Limitations
Weibel (Model A) [1]	1963	Symmetric dichotomy	Lung casts	23	No asymmetry No branching angles No gravitational angles
Horsfield (Delta Model) [2]	1971	Asymmetric dichotomy Self-consistency Branching angles	Lung casts	Up to 25	Inadequate for terminal inhomogeneities No spatial positions for branches
Phalen et al. [20]	1978	Unique branch identification Gravitational angles Record structural abnormalities	Lung casts	15–17	Data from only two casts
Yeh et al. [21]	1980	Lobar asymmetry Applicable to a lung portion	Lung casts	24	Data from only one cast Uncertain number of terminal bronchioles
Nelson et al. [22]	1988	Fractal geometry	Lung casts	NA	Limited to 2D
Kitaoka et al. [23]	1999	Consider the spatial position of the airways Rules to assign branch diameters, angles, lengths	Literature	14–16	More asymmetry than human lung Highly sensitive to host geometry and model parameters
Tawhai et al. [24]	2000	Growth of a bifurcating tree structure in the thoracic cavity	Imaging data	16–17	Diameters of branches with the same order are identical
Davoodi et al. [25]	2016	Lindenmayer system	Imaging data	23	Correlation between branching angle and diameter Does not consider the space occupied by other airways

### 2.1. Model Structures

**Figure 1:**
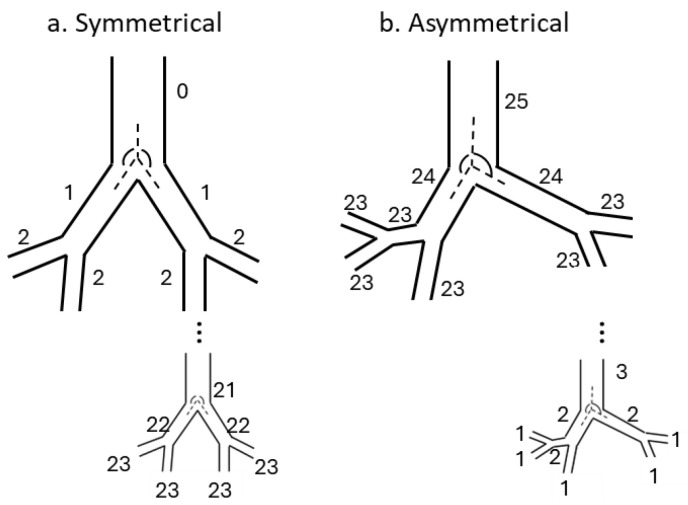
(**a**). Symmetrical branching airway tree. In this structure, each airway is identified by its generation number, starting from the trachea. (**b**) Asymmetrical airway tree structure proposed by Horsfield. In this model, each airway is identified by its order number, starting from the terminal bronchioles. Of note is the asymmetry introduced in the branching angles. The numbers in the figure indicate the generation order.

Symmetric models. In 1963, Weibel introduced the first mathematical model of the tracheobronchial tree [1]. Weibel’s Model A starts with the trachea (generation 0) and assumes all airways within the same generation are identical cylinders that bifurcate symmetrically into two branches (regular dichotomy). This pattern continues until reaching the alveolar sacs (generation 23, Figure 1a). The model, based on an adult human lung at three-quarters of its maximal inflation, ensures all branches within a generation share the same dimensions and branching angles. Despite providing detailed geometric representations, this model has limitations. It does not provide information on branching angles or angles of inclination relative to gravity, fails to encapsulate anatomical variations among or within lobes, and does not adequately account for asymmetry in daughter segment diameters, lengths, and angles. Furthermore, the assumption of a regular branching pattern, while suitable for healthy individuals, restricts its applicability in specific pathological conditions such as bronchoconstriction, where asymmetry and inhomogeneity are significant factors [23].

Asymmetric models. Asymmetrical models, unlike symmetrical ones, capture the intricate nature of human airway morphology by incorporating variations in airway diameters and branch angles. These models, grounded in actual measurements of human airway structures, offer a more realistic representation. As anatomical data became more detailed, these models achieved greater accuracy, especially in higher generations of airways. In the Horsfield model [2], starting from the terminal bronchiole (*n* = 1), the shortest path length was reached after eight dichotomous branches, whereas the longest path length was found after 25 branches. It introduced asymmetry at each bifurcation through a recursion index, varying throughout the bronchial tree. Limitations include a consistent degree of asymmetry at the same level in the tree, restricting heterogeneity at the terminal ends [26], and frequency domain analysis [27]. Phalen et al.’s model [20] considers the inclination of segments relative to gravity and records any abnormal structural features. Yeh et al.’s five-lobe lung model [21] introduced lobar asymmetry with variable path lengths among the five lobes, while maintaining symmetric branching within each lobe. This model, like Weibel’s Model A but without requiring a symmetrical tree structure, can be used to model specific portions of the lung.

Fractal geometry. The concept of fractal geometry, introduced by Mandelbrot in the early 1980s [28], was studied in relation to the structure of airways [22,29]. This concept was used to generate a model of airway morphogenesis, where growth followed a fractal pattern within a confined space defined by a clear boundary, such as the surrounding chest cavity. This model explained the irregularities observed in the terminal branches, as their characteristics were directly related to the need to fill the remaining space within the chest cavity. Weibel recognized that airway heterogeneity, self-similarity, and the absence of a specific scale were common features of fractal geometries [29].

**Figure 2:**
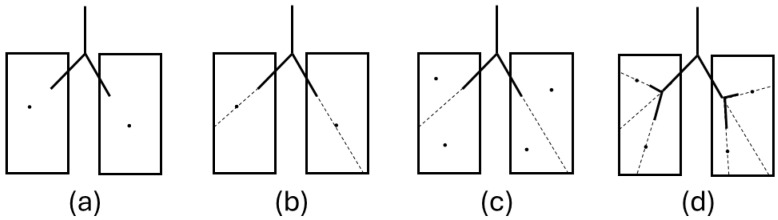
Airway tree generation in host volumes (represented in cross-section as rectangles) [24]. (**a**) Center of mass calculation: determine the center of mass (circles) for each volume. (**b**) Volume splitting: divide volumes using a plane (dashed line in cross-section) that includes the center of mass and the lobar bronchus vector. (**c**) Sub-volume center of mass calculation: calculate the centers of mass (circles) for the two sub-volumes. (**d**) Child branch generation: create child branches along lines from the end of the lobar bronchus to the centers of mass of the two sub-volumes.

Deterministic and stochastic models. While simplified geometries often overlook the spatial positions of the airways, some models have been developed to address this. Kitaoka and colleagues [23] developed an innovative approach to construct the bronchial tree in which the branching process is determined by the flow rate (Q), which, in turn, determines the diameter (d) of each branch. This model, which was expanded into a 3D model [30], generates a branching system within an organ, considering fluid delivery and uniform distribution of terminal branches. The model includes nine rules for defining the branching pattern and incorporates internal and external boundaries, such as the aorta and the heart, to establish a more anatomically consistent geometry for the generation of airways. However, it is important to note that while the outcomes of the Kitaoka model align reasonably with existing literature models, the resulting model was found to be more asymmetric than the actual human lung and sensitive to initial conditions [27]. Tawhai et al. [24] proposed a Monte Carlo-based model that constructs a 3D bronchial tree in each lobe, extending a previously proposed two-dimensional tree-growing algorithm [31]. They divided the space into sub-volumes using seed points and determined the branching angle and length based on the center of mass of the seed points (Figure 2). After constructing the entire structure, they randomly assigned diameters to all airways based on the Horsfield order. This model, further extended to include a CT-based geometry analysis and finite element models of the human and ovine bronchial tree [32], imposes a limit on branch angles and closely matches experimental data. Davoodi and Bozorgmehry’s model [25] used a stochastic rule-based method to generate the structure of the human bronchial tree using the Lindenmayer system (L-system). This rule-based technique allows for the definition of branch characteristics such as diameter, length, and branching angle, which can be calculated independently and in parallel. The model can construct airways in lungs with structural abnormalities and simulate the evolution of the airway structure based on lung volume growth over time. Further research by [33] expanded on the L-system by proposing multi-thread parallelism for simultaneous growth into bronchopulmonary segments. The system uses alphabetic rules for airway division and termination, with each module representing an airway’s geometrical characteristics, which are determined stochastically and independently. The model’s dimensionless, age-dependent parameters make it versatile for generating bronchial tree structures of various shapes and sizes.

### 2.2. Data Sources

Weibel (1963) and Raabe et al. (1976) [1,34] provided the two most comprehensive morphometric datasets for the tracheobronchial tree. These datasets encompassed various parameters pertaining to the structure of the airways, including their length, diameter, branching angle, and gravity angle. It is noteworthy that these models were based on morphometric studies conducted on individual lung casts. For each structural parameter, only a fraction of the airways was measured, and the arithmetic means were calculated.

Various approaches have been recommended to tackle the diversity in airway structure across individuals. Soong et al. [35] proposed using probability distributions to represent the lengths and diameters of airways, as well as the number and volume of alveoli. Yu et al. [36] developed a probabilistic lung model that integrates two random scaling factors to address variations in airway dimensions among different individuals. These scaling factors are specifically applied to the tracheobronchial region and the alveolar region of the lung. A fully asymmetric stochastic model was introduced by Koblinger and Hofmann [37,38,39], which allowed for the extension of the measured data from specific lungs to the overall adult population. In this model, the dimensions of the airways were described using probability density functions, and correlations among parameters were considered. All parameters were randomly selected from their respective statistical distributions, and different starting values for the random number generator would result in different bronchial tree configurations. This development significantly enhanced our understanding of airway geometry, although it still fell short of creating realistic image-based models.

### 2.3. Modeling Airway Diseases

Mathematical models often describe airways under pathological conditions using electrical, acoustical, and mechanical analogies. These models typically consider various parameters that depend on the specific multiscale morphological characteristics of the airways. This discussion focuses exclusively on assumptions related to morphological changes. A variety of theoretical models have been devised to simulate alterations in airway structure in obstructive lung disease. Wiggs et al. [40] examined the impact of airway wall thickening, loss of lung recoil, and airway smooth muscle shortening on the increase in airway resistance using a model of the human tracheobronchial tree. They found that moderate airway wall thickening, which has little effect on baseline resistance, can significantly affect airway narrowing caused by smooth muscle shortening, particularly if the wall thickening is localized in peripheral airways. They concluded that airway wall thickening and a loss of lung recoil can partially explain the airway hyper-responsiveness observed in patients with chronic obstructive lung disease and asthma. The anatomical information necessary for the model was obtained from histologic studies of the lungs of patients with asthma or COPD [41,42]. Gillis et al. [43] pioneered the concept of heterogeneous constriction, establishing an airway constriction distribution characterized by a mean and coefficient of variation, adhering to either a Gaussian or log-normal distribution. Their approach was applied to an asymmetric Horsfield model to predict flow distributions and their correlation with lung mechanical impairments. More recently, a stochastic airway diameter model has been proposed to provide a more anatomically precise representation of airways in asthma [44]. In the case of COPD, the human airway geometry has been depicted as a tube with a constriction along its length, mimicking the inflammation-affected region [45,46,47]. This constriction diminishes the cross-sectional area of the airways, consequently escalating the resistance of airflow in the impacted airway path [45]. Multiscale computational models of the airway have been developed to decode the intricate pathophysiological mechanisms in asthma and COPD by connecting cellular and molecular events to organ-level phenomena [48,49].

### 2.4. Airway Resistance in Respiratory Mechanics

The geometrical model of the airways is instrumental in determining airflow resistance, a fundamental aspect of respiratory mechanics. In the case of laminar and steady airflow, the resistance in a single branch can be approximated by using the Poiseuille formula, which shows that resistance (R) is directly proportional to the length of the tube (l) and inversely proportional to the fourth power of its radius (r): R = (8 μL)/(πr^4^), where μ is the gas viscosity [50]. The increased flow during exercise can cause significant turbulence in the proximal airways. This discrepancy is addressed by making empirical modifications to the formula in the tree’s early generations [50]. Further models have been proposed to account for the compliance of the airways and the air inertia [51,52].

Analogous to summing resistors in an electrical circuit, the aggregate resistance of the entire airway tree can be computed by adding the resistances of each individual branch. While it is accurate that a single smaller airway has a higher resistance than a larger one, the multitude of airways in the distal bronchial branching (those with a diameter of less than 2 mm) present resistances in parallel. Consequently, the overall resistance is diminished in the peripheral airways compared to the larger bronchi [53].

As evidenced by the Poiseuille formula, several factors influence airway resistance [50]. These include gas properties, such as density and viscosity, which affect the type of flow in the airways, with increased density leading to more turbulence and resistance, while increased viscosity promotes smoother flow and less resistance. The diameter of the airways, influenced by conditions like mechanical obstruction, dynamic compression, edema, mucosal, or smooth muscle hypertrophy, is crucial [51,54,55,56]. Indeed, the radius represents the most potent geometric determinant of resistance and links airway caliber directly to the function. This relationship is particularly important in obstructive lung diseases, where the lumen is compromised [55,56]. Smooth muscle tone, affected by factors like bronchodilators, sympathetic nervous system agonists, bronchospasm, irritants, and parasympathetic nervous system agonists, also impacts airway diameter and resistance [54].

## 3. Image-Based Anatomical Models

### 3.1. CT Imaging of Lung Casts

The initial exploration of realistic geometries was achieved by leveraging a blend of lung casts and CT imaging to construct digital models, which can be replicated or utilized in numerical modeling research. The Visible Human Project [57], supported by the National Institutes of Health/National Library of Medicine (NIH/NLM), provided high-resolution CT scans of cadaver casts of both genders. Clinkenbeard et al. [58] pioneered the creation of a detailed hollow airway model encompassing the first five airway generations, employing rapid prototype techniques.

Schmidt and colleagues [59] crafted a digital lung model, encompassing 17 generations but excluding the complete trachea, by scanning a rubber cast of lungs from an autopsy and applying image processing algorithms to segment the bronchi. From the binary representation of the bronchial tree, two distinct models were derived. One was a surface representation of the segmented volume, while the other was a graph representation detailing the branching topology, diameter, and length of each branch. This comprehensive graph facilitated the reconstruction of a simplified, tube-like surface representation of the bronchial tree’s geometry. A statistical examination of the bronchial tree’s diameters and lengths confirmed the model’s pronounced asymmetry and multifractal properties. To conduct optical and deposition measurements, Lizal et al. developed five unique airway geometries that extended to the seventh bifurcation. These geometries, proposed within the framework established by Schmidt et al., encompassed both realistic and semi-realistic representations, incorporating variations with and without an oral cavity [60].

### 3.2. Patient-Specific Models

Patient-specific models address inter-subject variability and potential pathological conditions. Advances in imaging and computing technologies enable detailed anatomical measures, along with functional imaging like ventilation and perfusion. Analyzing airway geometry involves segmenting the bronchial lumen and wall from thoracic scans, a challenge due to the complex tree-like structure with numerous branches of varying sizes and orientations. Thin walls often fall below scan resolution and can be obscured by partial volume effects, noise, or pathological processes. In this section, we offer a comprehensive overview of image segmentation algorithms, with a particular emphasis on the latest deep learning-based approaches, and discuss mesh generation techniques tailored for patient-specific models.

#### 3.2.1. Airway Segmentation

##### CT Imaging

**Figure 3:**
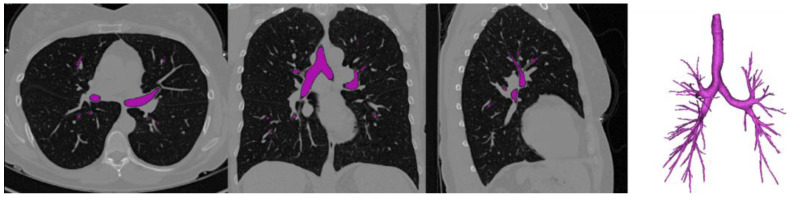
Axial, coronal, and sagittal views of a CT scan from a healthy volunteer. The airways have been segmented using a 3D confidence-connected region-growing algorithm. The original CT data are from [82], acquired at Washington University in St. Louis, MO, USA.

Numerous techniques have been proposed over recent decades for automated reconstruction of the tracheobronchial tree, aiding in measuring abnormalities (Figure 3). In CT scans, airways appear as tubular structures with varying intensities, surrounded by a background that may indicate lung parenchyma or vessels. We categorized automatic airway segmentation into traditional and machine learning methods.

Traditional methods. Gray-level-based region-growing techniques are early methods for airway segmentation from CT images, using seed points within the airway tree and expanding the region based on intensity or gradient similarity [61]. While these methods accurately identify central bronchi, they struggle with smaller branches and often result in leakage into the lung parenchyma. Various strategies have been used to address this issue, such as front propagation, sharpening pre-processing, branch-specific thresholds, and fuzzy connectivity, as widely reviewed in the literature [3,62]. In the EXACT’09 airway extraction challenge, most algorithms (11 out of 15) used region-growing approaches, but there were limitations in accurately capturing smaller branches and false-positive errors [63]. Knowledge-based techniques use anatomical knowledge and contextual relationships to reconstruct the bronchial tree. Sonka et al. identified large airways through region-growing approaches and analyzed small airways based on their association with pulmonary vessels [64]. Park et al. applied fuzzy logic evaluation brightness, adjacency, and wall presence [65]. Fan et al. used a morphological approach focused on no loops and abrupt changes in direction at branching points [66]. These methods may not be suitable for unusual or pathological anatomy. Morphological techniques identify 3D airway structures through a two-step process: identification using morphological operations followed by differentiation of genuine airways using 3D connections and shape attributes [67]. Additionally, tubular structure detection and analysis of local derivatives have been used for segmenting airways [68]. However, continuous detection of the airways is required for proper reconstruction, and these methods are usually time and computationally consuming [3].

Machine Learning and Deep Learning Approaches. Airway segmentation methods based on machine learning classifiers have emerged for reconstructing the airways, either for voxel-wise airway classification or to remove false-positive airway candidates from a leaky segmentation, enhancing the sensitivity and specificity of existing methods. By leveraging machine learning, it becomes possible to develop probability distributions that analyze multiple features simultaneously to determine the likelihood that a given airway candidate accurately represents a true airway. Lo et al. [62,69] proposed a kNN classifier to detect airway regions and distinguish them from surrounding structures. Instead of using conventional image intensity features, they used probability distributions derived from local image descriptors for segmentation, reducing leakages and improving the segmentation of smaller branches. The method was later improved by incorporating vessel orientation similarity measures and an airway appearance model [70]. Inoue et al. [71] proposed a machine learning approach involving various steps: Hessian analysis to identify branches, thresholding for detecting tubular structures, and AdaBoost machine learning for reducing false positives. The remaining airways are used to generate the airway tree by considering orientation, intensity, and scale. Prim’s method is applied to create a minimum spanning tree that provides valuable information about the geometry, centerlines, and radii of each airway segment. Finally, 3D Graph Cuts are used to segment the airway regions [72]. Nevertheless, their effectiveness is heavily reliant on the image features employed for classifier training, which can be a time-consuming process due to the computation required for extracting these features.

In recent times, cutting-edge techniques for medical image segmentation have used deep learning, and in particular convolutional neural networks (CNNs) [73,74,75,76,77]. Charbonnier et al. [73] proposed a method for addressing airway leakage using convolutional neural networks (CNNs). They treated the problem of leakage as a classification task: a collection of 2D patches that represented the 3D appearance of the airway along its centerline are extracted and classified using a CNN, allowing for the removal of leaked segmentation branches. Yun et al. [74] applied the 2.5D CNN approach, which processes the three perpendicular 2D slices around each voxel, to perform voxel-wise airway classification. The U-Net architecture has found extensive application in airway segmentation [78,79,80]. U-Net architecture, with its ability to process complete images in a single pass, generates segmentation maps directly. These techniques offer comparable results with previous approaches, with improved sensitivity for leakage and better specificity against false positives. In the airway segmentation challenge task at the 4th International Symposium on Image Computing and Digital Medicine (ISICDM 2020), 9 out of 12 teams chose U-Net or its variants, which included attention mechanisms and multiscale feature fusion [81]. In a recent development, Garcia-Uceda et al. [78] present a fully automatic and end-to-end [61,73,79] optimized airway segmentation method for thoracic computed tomography, based on the U-Net architecture, specifically a 3D U-Net, which allows the processing of large 3D image patches, often encompassing full lungs, in a single pass through the network. This makes the method simple, robust, and efficient. The method has been validated on three datasets with very different characteristics and various airway abnormalities, and it has been shown to extract highly complete airway trees with few false-positive errors on scans from both healthy and diseased subjects. On the EXACT’09 test set, their method achieved the second-highest sensitivity score among all methods that reported good specificity.

##### Segmentation of Pathological Bronchial Trees

A limited number of studies specifically address airway segmentation in the case of pathological bronchial trees. Benchmark datasets have been created to aid in the advancement of data-driven techniques and to allow for a comprehensive assessment of the effectiveness of new algorithms [64,76,83]. However, these datasets contain a limited amount of data related to airway abnormalities. The recent Multi-site, Multi-domain Airway Tree Modeling (ATM’22) challenge, organized as a part of the MICCAI 2022 conference, released a large dataset that encompassed both screening and COVID-19 patient data, but few patients with severe pathology affecting the airway tree anatomy were included [83].

Only a few papers measure segmentation performance on scans from both healthy and diseased subjects. On severe COPD patients, where airway detection can be more challenging due to large low-attenuation areas, authors reported a significant decrease in sensitivity and reliability, with varying execution times despite the optimization [74,78]. Irving and colleagues focused on bronchoconstriction, which allowed for the segmentation of airways beyond areas of obstruction to improve the efficiency of automated airway analysis. Their approach involved utilizing airway topology and shape to identify disconnected airway segments [4]. Cystic fibrosis has been associated with an increased presence of segmented airway branches, potentially attributed to the expansion of peripheral airways caused by CF bronchiectasis. This phenomenon facilitates their identification on CT scans [78,84]. A few studies have presented techniques for automatically matching airway branches with their corresponding artery to extract measurements of wall thickness and bronchiectasis [85,86]. Limited research has focused on airway recognition in the case of non-traction bronchiectasis [87].

##### Magnetic Resonance Imaging (MRI)

Traditional MRI. Advancements in MRI technology and specific imaging sequences have enabled the radiation-free imaging of the lungs, comparable to CT scans. This is particularly beneficial in cases where high repeatability of examinations is necessary or when dealing with young patients. However, traditional MRI of lung structures faces challenges in terms of image quality and resolution due to the low proton density in lung tissue. The presence of artifacts from respiratory and heart motion further complicates the imaging process, leading to longer acquisition times [88]. Ivanovska et al. [89] focused on MRI trachea segmentation to evaluate tracheal stenosis during surgical interventions for pathological conditions.

Hyperpolarized gas MRI. The utilization of contrast agents has partially mitigated the challenges of traditional MRI. For instance, Lewis and colleagues [90] utilized hyperpolarized ^3^He MRI to accurately measure the diameters of the first five generations, yielding consistent results when compared to the expected dimensions based on Weibel’s model. Nevertheless, there is a lack of specific segmentation methods for MRI data in the reconstruction of the airway tree, except for the work by Wang et al. [91]. Lewis et al. [90] employed a combination of commercially available software and manual segmentation to successfully segment the upper respiratory tract. Peterson et al. [92] achieved a rough segmentation down to the third generation by using hyperpolarized helium-3 MRI in conjunction with dynamic 3D radial acquisition.

Ultrashort Echo Time (UTE) MRI. Despite advancements in imaging and segmentation techniques, MRI-based reconstruction of the tracheobronchial tree remains a challenge. However, Radial Ultrashort Echo Time (UTE) MRI has shown promise. This technique samples the rapidly decaying pulmonary MR signal much earlier than conventional MRI, yielding images with resolution and proton-density image intensity approaching that of CT [93,94]. Interesting results in the analysis of the airways have been reported in neonatal tracheomalacia [95] and Obstructive Sleep Apnea [96]. Genkin et al. [97] demonstrated the feasibility of UTE MRI airway segmentation from the trachea-to-tertiary airways in pediatric patients across a range of diseases. It is worth mentioning the combination of static high-spatial-resolution MRI with high-temporal resolution 4D MRI to create virtual moving airway surfaces [98]. These advancements in UTE MRI technology have opened new avenues for the reconstruction of the tracheobronchial tree, although further research is needed.

#### 3.2.2. From Segmented Airways to Mesh Generation

**Figure 4:**
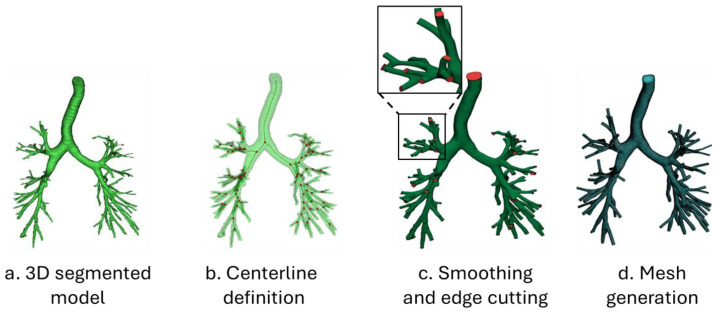
The steps required to achieve an accurate mesh model. (**a**) Airways segmented with a 3D confidence-connected region-growing algorithm; (**b**) centerline delineation; (**c**) global and local smoothing and edge cutting performed in 3-Matic (Materialized NV, Belgium) and Ansys SpaceClaim (Ansys Inc., Canonsburg, PA, USA); and (**d**) mesh generation in Ansys Fluent (Ansys Inc., Canonsburg, PA, USA). The original CT data are from [112].

Surface geometries from segmented airways in CT images are initially unfit for computational techniques due to their inherent roughness and noise. The main steps to achieve a CFD-compliant mesh are resumed in Figure 4.

Centerline extraction. Airway surfaces can be modeled based on a centerline curve. The primary approach defines airway walls as 2D cross-sectional contours along the centerline. The curvilinear 1D centerline is extracted using a skeletonization algorithm, such as the Voronoi skeleton [9] or the thinning algorithm [99]. The Voronoi skeleton method constructs a Voronoi diagram from the boundary voxels. In contrast, the thinning algorithm methodically erodes the object’s voxels layer by layer, maintaining its topological and geometrical properties, and results in centrally located skeletal lines with a single voxel width. To ensure precise categorization of skeleton points, including branch points and bifurcations, a smoothing process can be applied to the skeleton point cloud to strike a balance between smoothness and displacement [100]. Specific strategies have been designed to accurately depict airway bifurcations [100,101]. Hegedus [101] introduced a mathematical representation of a morphologically accurate airway bifurcation, employing their methodology to amalgamate several bifurcations into diverse airway geometries. A notable attribute of the model is its enforcement of a seamless transition between airways, coupled with rounding at the carina.

Smoothing and edge cutting. Surface smoothing is a crucial step in accurately processing the geometry [102], aiming to eliminate surface noise while preserving intrinsic features. In CFD simulations, unsmooth geometries can distort flow pattern representations, impacting airflow and particle transport predictions in airway models. Conventional smoothing approaches, such as the Laplacian and Taubin methods, have been used in CFD studies [103,104]. Despite extensive research on mesh denoising, including filter-based methods [105], optimization-based approaches [106], and feature-preserving strategies [107], geometric feature extraction in airway models remains challenging. Data-driven techniques, such as the unsupervised airway-mesh-smoothing learning (AMSL) method, leverage machine learning and deep learning [103,108,109]. However, these learning-based mesh denoising methodologies often face two main drawbacks: a dependence on single-modal geometric representations, limiting their ability to capture the multifaceted attributes of meshes, and a lack of effective global feature aggregation, hindering their comprehension of the mesh’s comprehensive structure. During geometry preparation prior to mesh generation, it is essential to maintain regular and circular shapes of the inlet and outlet surfaces. This is crucial for defining boundary conditions and ensuring a fully established flow as it enters and exits the simulation domain. Various strategies have been proposed to trim side branches, including manual pruning [110] and path length thresholding [99]. By achieving a uniform velocity profile across the inlet and outlet areas, numerical convergence is promoted, thereby enhancing the accuracy of the results and the path length thresholding. Achieving a uniform velocity profile across the inlet and outlet areas promotes numerical convergence and enhances result accuracy.

Mesh generation. The mesh model must faithfully depict the anatomical structure, free from any imperfections that could result in air leakage or airflow blockages. The effectiveness of the resulting discretized domain, in terms of how well it captures the airway geometry and its suitability for resolving airflow patterns, is contingent upon the quality of the CT images, the precision of segmentation (the 3D reconstruction process of the airways), the meshing algorithm utilized, and the thoroughness of testing mesh refinement to ensure convergence of the numerical solution [111].

While various research studies have leveraged commercial software for mesh creation, this often requires extensive manual adjustments to correct the resulting meshes [113,114,115]. Alternatively, open-source software programs like 3D Slicer or MeshLab can generate meshes from 3D medical images [116,117]. These programs employ surface reconstruction algorithms, such as marching cubes (MCs), to accomplish this task. However, when applied to lung airway geometry, these algorithms do not inherently include explicit constraints to prevent holes and airway blockages. Recent advancements in the field include the work of Lauria et al. [100], who developed an automatic method for generating triangulated meshes of pulmonary airways from segmented lung 3DCTs for computational fluid dynamics. Their method creates a CFD-compliant airway mesh free from airflow blockages and leaks, issues often arising from errors in conventional meshing techniques. Starting from the central skeleton, the method identifies airway branches and bifurcations, enabling an automated meshing process that considers their anatomical properties [100]. Ortiz-Puerta et al. [118] developed a computational framework using non-uniform rational B-splines (NURBSs) to create geometric representations of the airway tree, offering flexible and customizable lumen shapes. This approach, known as Snakes Isogeometric Analysis (SIGA), has demonstrated high accuracy in representing the surfaces of both healthy and COPD airway trees.

Generating airway meshes from CT images can be computationally demanding, especially for methods involving complex algorithms or high-resolution images. This demand can restrict their use in clinical settings with limited computational resources. Accuracy is another major challenge, as segmentation errors can lead to inaccuracies in the resulting airway mesh, causing airflow blockages and leaks. Many current methods require time-consuming manual adjustments, which can introduce bias and may not be feasible or accurate for complex structures. To enable the use of computational fluid dynamics simulations in clinical settings, a robust, automated, and CFD-compliant airway mesh generation technique is needed. However, developing such methods presents a significant challenge, underscoring the need for continued research to enhance the efficiency, accuracy, and usability of these methods.

## 4. Hybrid Models

**Figure 5:**
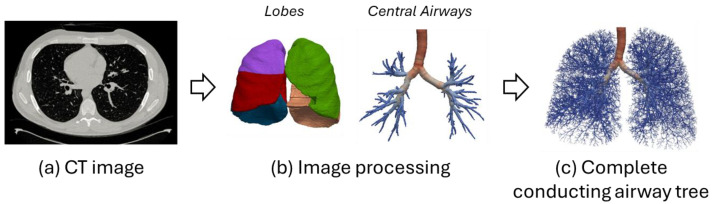
Complete patient-based airway model to the acinar level obtained through an image analysis and modeling pipeline. (**a**) CT image; (**b**) lobes and central airways segmented from the CT image; and (**c**) airway tree beyond CT resolution generated with the lobe-filling algorithm. The tree is color-coded by airway radius. Adapted from [17].

Hybrid methods that combine CT imaging and mathematical models have been proposed to create more accurate models of the airways. While morphometric lung models are useful for representing distal lung geometry, they require several approximations for CFD analysis and do not account for subject-specific geometric features and pathological conditions. On the contrary, image-derived lung geometries enable patient-specific modeling but are often limited to a few generations beyond the trachea due to the small scale of the distal airways. To overcome this limitation, hybrid lung models have been proposed that combine CT-derived patient-specific geometry with a tree model based on a deterministic algorithm. This approach enables the generation of the airway tree structure from the trachea to the terminal bronchiole level, retaining subject-specific geometric features up to the CT-resolved airway and providing CFD-compliant geometry for distal airways. Physiological pressure-driven flow is simulated by applying patient-specific boundary conditions, allowing the methodology to be readily adapted to any patient-specific pathological condition and enabling detailed CFD analysis for flow distribution in the distal airways. Several studies have established methods to create a fully resolved 3D mesh from the trachea to any terminal bronchioles of interest, allowing for the simulation of fluid and particle transport from the model entrance to the level of lung parenchyma [16,119,120]. The airway tree beyond CT resolution is generated by a volume filling method (VFM) [24,32] that takes the skeleton of the 3D CT-resolved central airway tree in a human subject and then generates a tree to fill the entire volume within the subject’s respective five lobes (Figure 5). The resulting airway trees are consistent with measurements from airway casts and imaging studies, although they are only specific to the subject’s lung lobe shapes and orientation of their central airways. The hybrid model allows the application of physiologically relevant boundary conditions in lieu of simplistic approximations used in previous studies. Yoon et al. [121] further enhanced these models by developing a 1D CFD model with dynamic airway geometry, considering airway wall compliance and acinar dynamics.

## 5. Applications

Computational models of the airways have diverse applications across different disciplines, enabling breakthroughs in fields such as medicine, pharmacology, and environmental science.

### 5.1. Inter-Subject Geometry Variability

In a key study, Kim et al. [122] used the Weibel and Kitaoka [123] models to investigate airflow and aerosol deposition. They found that geometric variations greatly influenced deposition patterns. While the Weibel model showed symmetrical airflow, the Kitaoka model displayed asymmetry. However, changes in flow rates had similar effects across both models. Espinosa-Moreno et al. [124] used numerical simulations to explore how changes in the bifurcation angle and carina rounding radius of the lower human airways impact airflow dynamics. They found that these parameters significantly influenced airflow development, affecting aspects like velocity profiles, pressure drop, flow patterns, and wall shear stresses [124].

Understanding the importance of inter-subject variabilities, Poorbahrami et al. [125] conducted a study on three adult females to understand how anatomical variations in airways affect airflow and particle deposition. Their research highlighted the importance of considering inter-subject variability in lung modeling for accurate clinical predictions. The impact of morphological variations in mouth-to-glottis configuration parameters on airflow and inhaled particle transport characteristics in the tracheobronchial tree has also been investigated [126].

Numerous studies have examined airflow and aerosol deposition variations across age groups using idealized, age-scaled airway models [127,128]. In a notable contribution, Poorbahrami et al. [126] incorporated CT-based airway geometries and realistic respiratory waveforms [125] across different age groups, finding an inverse correlation between air speeds and airway resistances.

### 5.2. Medical Applications

Geometrical models of the airways have significantly contributed to quantifying and visualizing airway remodeling. These models have diverse applications in the medical field, from understanding disease mechanisms to predicting disease progression, and from facilitating personalized treatment plans to strategizing drug delivery.

Insight into the structure–function relationship. Complete conducting airway tree models have occasionally been used in a patient-specific setting in conjunction with functional imaging data such as positron emission tomography (PET) and hyperpolarized gas MRI to improve our understanding of the structure–function relationship in lung disease [129,130]. As previously detailed, various theoretical models have been developed to mimic changes in airway structure. These models aim to assess flow and pressure distribution in healthy individuals and patients with obstructive lung diseases. The advent of CT imaging in airway modeling has facilitated the creation of innovative models that integrate dynamic distortions observed in the airways throughout the respiratory cycle [131,132]. By integrating computational models and CT-based dynamic deforming airways, it has been possible to enhance augmented hysteresis of the pressure–volume curves and a higher workload in the asthmatic group [121,133]. Patient-specific simulations of aerosol dosimetry in asthma have revealed high inter-patient variability in total and regional deposited particle concentrations, underscoring the importance of patient-specific geometrical models [134]. CFD simulations with realistic airway models in patients with mild or moderate asthma have shown a good agreement with functional imaging and in vivo experimental data [135,136]. It is worth noting that CT image-based airway models have also allowed investigation of structure–function relationships in other diseases, such as left pulmonary artery sling [137] and tracheal bronchus [138], showing that these specific structural features of these conditions present individualized flow attributes.

Surgical planning. Computational models have proven invaluable in surgical planning, particularly in complex interventions. By simulating various surgical scenarios, clinicians can gain a better visualization and understanding of potential outcomes, leading to improved surgical precision, reduced complications, and enhanced patient outcomes. Surgical interventions have been widely investigated through computational models in the upper airway, such as maxillomandibular advancement surgery [139,140] or OSA treatment [141]. Hamilton et al. [142] demonstrated the long-term viability of a decellularized tissue-engineered trachea within a child by determining flow, velocity, and airway pressure drops through CFD simulations based on CT-reconstructed airways. The fluid dynamics effects of pulmonary lobectomy have been recently studied, focusing on comparing the pre- and postoperative conditions [143,144]. In this context, CFD analysis was used to analyze flow dynamics in lung cancer patients to evaluate tumor impact on flow parameters and lobar distribution and to predict postoperative forced expiratory volume in 1 s using patient-specific airway models reconstructed from CT images [112].

Obstructive Sleep Apnea (OSA). Although the focus has been primarily on the upper airways, a considerable body of research has been dedicated to simulating the process of airway obstruction in OSA [145,146]. In adult populations, CFD analysis has been employed to distinguish OSA patients from non-OSA individuals [147], predict potential sites of collapse [148], investigate the impact of various breathing routes on airway collapsibility [149], and examine the alterations in morphology and internal airflow after the use of oral appliances in patients with OSA [150]. These studies have provided valuable insights into the mechanisms of airway obstruction and the effectiveness of therapeutic interventions.

However, there is a relative scarcity of similar studies conducted in pediatric populations [151,152]. Pediatric OSA poses unique challenges due to anatomical and physiological differences from adults, with research focusing on developmental airway obstruction and the effectiveness of treatments like adenotonsillectomy and continuous positive airway pressure (CPAP) therapy [153,154]. CFD analysis in pediatric populations can enhance understanding of OSA pathophysiology [152] and improve diagnostic and therapeutic accuracy [155,156]. Future studies should use advanced imaging and patient-specific modeling to better understand the unique characteristics of pediatric OSA and develop targeted interventions.

Diagnosis. Airway models, leveraging computational techniques and machine learning algorithms, have been instrumental in providing non-invasive, cost-effective, and accurate diagnostic methods for various diseases. In the context of understanding the impact of small airway obstructions on overall airflow, Hariprasad et al. [157] used patient-specific geometry to investigate the influence of these obstructions on the flow patterns of the upper airways. For patients with COPD, Hu et al. [11] proposed a method for locating small-airway obstructions based on CFD and CNNs on a 3D tracheobronchial tree model. The CNN models, trained on airflow velocity contours, could classify lung obstructions with over 95% accuracy, showing that this method could revolutionize early diagnosis of lung obstructions. In the context of infectious diseases, specifically COVID-19, Qiu et al. [158] utilized CFD to calculate airway resistance based on airway anatomy and airflow rate, finding a significant correlation between airway resistance at admission and the prognosis of COVID-19 patients, suggesting its potential as a diagnostic index. In the field of oncology, Xi et al. [12] introduced a tool that uses exhaled aerosol distribution to locate malignant sites in a non-invasive and low-cost way. The tool was tested in an image-based lung model with varying stages of a bronchial squamous tumor. The results show that morphometric measures of the exhaled aerosol pattern can detect and monitor pathological states of respiratory diseases in the upper airway.

### 5.3. Pharmacological Studies

Airway geometries are pivotal in pharmacological studies, especially in the development and testing of respiratory drugs. The geometry of airways significantly influences the delivery and deposition of inhaled drugs, thus accurate models are instrumental in designing effective drug delivery systems. Studies have explored the impact of a realistic inhalation profile on a range of drug particle sizes and morphologies [159,160] and inhalation flow rates [14,160,161,162,163]. Notably, Kadota et al. [162] used a CT-based realistic human airway model to analyze the effects of six airflow patterns on the behavior of inhaled particles. They found that different inhalation flow rates influenced the deposition of inhaled particles, with distinct airflow tendencies in the right and left bronchi, suggesting that applying their analysis to individual lungs could lead to patient-specific dry powder formulations.

Airway models also serve as platforms for simulating various respiratory diseases and testing drug delivery effectiveness. For instance, these models have been used to study the effects of drugs on airway constriction in asthma [164,165]. Using a whole-lung CFD modeling approach, it was demonstrated that inhaled particles provide a low drug dose per unit surface area to small airways [166] and that localized lung diseases and inhaled antibiotic concentrations are highly correlated [167]. With the advent of personalized medicine, there is a growing interest in developing patient-specific models of the airways to predict individual responses to different drugs, leading to more personalized and effective treatments [168].

Recently, the Magnetic Drug Targeting (MDT) technique has been explored for targeted drug delivery to specific regions in realistic human airway models [169]. The researchers analyzed the impact of magnetic source position, magnetic field intensity, and magnetic particle size on particle deposition efficiencies, aiming to identify the optimal combination for effective drug delivery.

### 5.4. Environmental Applications

Computational airway models also find application in environmental sciences. They assist in understanding pollutant dispersion within the airways, respiratory system responses to pollutants, and the impact of environmental exposures on respiratory health. Such models aid in formulating preventive measures, environmental policy regulations, and assessing potential health risks.

For instance, Paul et al. [170] utilized a CT scan-based realistic airway model to investigate the deposition fraction of cigarette smoke particles of different sizes. In a following study [171], the same authors explored the airflow characteristics and the deposition of suspended particulate matter (PM2.5 and PM10) in air with an unhealthy air quality index, using a realistic geometric model of human airways. Further research revealed a correlation between increased disease severity and higher particle deposition in the airways of children with asthma, suggesting that asthmatic children are more susceptible to the impact of particulate air pollution [172]. Rahman et al. [173] used CFD to investigate the transportation and deposition of pollutant particles of various densities and sizes, ranging from nano- to micro-scales. Dong et al. discussed the health risks associated with exposure to ambient air pollution, particularly in the deep lung regions. They used an extended lung airway model to study the deposition of particles, ranging from 100 nm to 3.0 μm, corresponding to the major size spectrum of coarse diesel exhaust. Their findings showed that particle deposition in the respiratory airways is sensitive to inhalation flow rates, with higher breathing rates scattering particles over the lower respiratory airway [174]. In conclusion, the forces that dictate particle deposition in the lungs depend on the particle’s size, with impaction being the primary mechanism for larger particles, sedimentation for medium-sized particles, and diffusion for ultrafine particles. These mechanisms are subject to influence by various factors, such as the morphology of the lung, breathing patterns, and the geometry of the airway.

In a broader context, CFD methods have been instrumental in examining pathogen transmission [175], particularly in relation to the factors influencing their spread across various environments and the impact of airflow on this process. A recent review encapsulated the CFD-based research conducted on the COVID-19 pandemic [176]. This includes studies on transmission in confined spaces under different ventilation conditions, as well as the effects of social distancing and mask usage on reducing the dispersion of virus-laden microdroplets. These reviews highlighted the potential of these models in mitigating disease spread and highlighting the critical role of environmental factors in disease transmission.

## 6. Discussion

In this study, we explored a variety of realistic geometrical representations of the airways, ranging from initial simplified mathematical models to individual-specific geometries derived from images.

Mathematical models were initially developed to simplify and analyze the complex structure of the tracheobronchial tree when imaging techniques and computational power were insufficient. These models gradually tackled the problem by increasing the complexity, starting from a symmetrical bifurcating pattern and advancing towards a more comprehensive and accurate asymmetric approach. Many of these models were based on existing anatomical knowledge or observations of lung casts. They relied on a set of assumptions that, in most cases, are still considered reasonably acceptable today, especially for the larger airways under physiological conditions. It is noteworthy that some of these models continue to find applications in various research fields [177,178,179]. However, the main limitation of geometrical models is their inability to account for patient-specific or pathology-induced alterations. This limits their clinical applicability compared to image-based models. To address this limitation, some geometrical models have been integrated with image-based models to describe higher-order airways. It is important to note that most of these models do not consider the spatial relationship with the surrounding lung structures [48]. This review highlighted a scarcity of studies that delve into the mathematical modeling of pathologies, with a predominant focus on broncho-constrictive diseases. The use of these models is primarily geared towards research purposes, as their ability to describe clinical scenarios is severely constrained by the assumptions made regarding the initial geometry and the imposed pathological changes, which are frequently arbitrary or derived from broad ex vivo analyses. For instance, it is noted that asthma-related airway changes are part of a complex network of interconnected disease processes and that single mechanisms may not fully explain the system’s behavior or the emergence of disparities in the airways’ structures and functions [180,181].

Since the 1990s, the rapid advancement in medical imaging hardware and techniques has been a catalyst for research towards image-based reconstruction models. These models, derived from imaging, can generate patient-specific models, thereby addressing inter-subject variability and potential pathological conditions. In this context, the segmentation of the bronchial tree from thoracic scans is deemed a crucial step. Despite the significant progress made with deep learning techniques, there is still a need for improvements in the use of segmentation within routine clinical practice, particularly in terms of accuracy, specificity, and overall quality. A significant issue arises from the fact that regardless of the segmentation method used, numerous small airways are either missing or inaccurately identified. Moreover, there has been limited focus on applying segmentation techniques to scans from patients with pathological conditions.

The application of image-based models is constrained by the need for rigid and regularly shaped structures, which align with the traditional engineering methods of fluid and structural mechanics. Generating airway meshes from CT images can be computationally demanding, which can limit their use in clinical settings. Furthermore, accuracy is a major challenge, as segmentation errors causing airflow blockages and leaks require time-consuming manual adjustments. Current research in CFD-compliant airway mesh generation techniques may bridge this gap in clinical applications [100]. However, it is important to note that these techniques are still in their developmental stages and require further refinement and validation.

## 7. Conclusions

This article provides a critical examination of the most prominent methodologies proposed for accurately representing the tracheobronchial tree. It begins with an exploration of mathematical approaches before transitioning to more contemporary image-based techniques. The importance of the mathematical models should not be underestimated, not only in relation to the historical and technical context in which they were developed but also for their applicability in modern engineering approaches where a degree of approximation or simplification is not only accepted but also desirable. Conversely, image-based models, emerging from advancements in imaging research, provide a unique opportunity for personalized modeling and treatment. Our review provides a novel integrative perspective by highlighting how the combination of imaging techniques with mathematical models creates unique opportunities for developing patient-specific models. When combined with functional imaging, these models could serve as a bridge to comprehend the interplay between structural and functional pulmonary impairments. Despite their limitations, they can be considered the most tangible and practical means of achieving future clinical applications.

The clinical implications of this approach are significant, as personalized models can enhance diagnostic accuracy, enable earlier disease detection, monitor progression, and evaluate treatment effectiveness, leading to more tailored and effective patient care.

## Data Availability

No new data were created or analyzed in this study. Data sharing is not applicable to this article.
